# Amyloid-β Associated Cortical Thinning in Clinically Normal Elderly

**DOI:** 10.1002/ana.22333

**Published:** 2011-03-17

**Authors:** J Alex Becker, Trey Hedden, Jeremy Carmasin, Jacqueline Maye, Dorene M Rentz, Deepti Putcha, Bruce Fischl, Douglas N Greve, Gad A Marshall, Stephen Salloway, Donald Marks, Randy L Buckner, Reisa A Sperling, Keith A Johnson

**Affiliations:** 1 Department of Radiology, Martinos Center for Biomedical Imaging, Massachusetts General Hospital, Harvard Medical SchoolBoston, MA; 2 Athinoula A. Martinos Center for Biomedical Imaging, Massachusetts General Hospital, Harvard Medical SchoolBoston, MA; 3 Department of Neurology, Brigham and Women's Hospital, Harvard Medical SchoolBoston, MA; 4 Neurology, Martinos Center for Biomedical Imaging, Massachusetts General Hospital, Harvard Medical SchoolBoston, MA; 5 Computer Science and Artificial Intelligence Laboratory, Electrical Engineering and Computer Science, Health Science and Technology, Massachusetts Institute of TechnologyCambridge, MA; 6 Department of Neurology, Warren Alpert Medical School, Brown UniversityProvidence, RI; 7 Memory and Aging Program, Butler HospitalProvidence, RI; 8 Tufts School of MedicineBoston, MA; 9 Department of Psychology and Center for Brain Science, Harvard UniversityCambridge, MA; 10 Psychiatry, Martinos Center for Biomedical Imaging, Massachusetts General Hospital, Harvard Medical SchoolBoston, MA; 11 Howard Hughes Medical InstituteCambridge, MA

## Abstract

**Objective:**

Both amyloid-β (Aβ) deposition and brain atrophy are associated with Alzheimer's disease (AD) and the disease process likely begins many years before symptoms appear. We sought to determine whether clinically normal (CN) older individuals with Aβ deposition revealed by positron emission tomography (PET) imaging using Pittsburgh Compound B (PiB) also have evidence of both cortical thickness and hippocampal volume reductions in a pattern similar to that seen in AD.

**Methods:**

A total of 119 older individuals (87 CN subjects and 32 patients with mild AD) underwent PiB PET and high-resolution structural magnetic resonance imaging (MRI). Regression models were used to relate PiB retention to cortical thickness and hippocampal volume.

**Results:**

We found that PiB retention in CN subjects was (1) age-related and (2) associated with cortical thickness reductions, particularly in parietal and posterior cingulate regions extending into the precuneus, in a pattern similar to that observed in mild AD. Hippocampal volume reduction was variably related to Aβ deposition.

**Interpretation:**

We conclude that Aβ deposition is associated with a pattern of cortical thickness reduction consistent with AD prior to the development of cognitive impairment. ANN NEUROL 2010;

The possibility of disease-modifying therapies for Alzheimer's disease (AD) has motivated the development of biomarkers that reflect underlying pathologic processes. The sequence of pathologic events in AD likely begins many years, perhaps decades, prior to the development of symptoms.^1^, ^2^ Amyloid-β (Aβ) deposition appears early in the disease, prior to symptoms, and then plateaus as clinical dementia emerges.^3^–^6^ In contrast, neurodegeneration, including loss of synapses, neurons, and arborization, results in brain atrophy that worsens in parallel with cognitive decline.^2^, ^6^, ^7^ The principal early sites of Aβ deposition are neocortical, typically in the parietal and frontal regions,^4^, ^8^, ^9^ whereas the sites of early atrophy include the medial temporal regions.^2^, ^6^, ^10^, ^11^ Here we relate these 2 phenomena in vivo in clinically normal (CN) older individuals and in clinically established AD patients, in order to determine the correspondence between levels of Aβ deposition and of atrophy.

It is now possible to observe the relation between Aβ deposition and atrophy in vivo with positron emission tomography (PET) imaging using Pittsburgh Compound B (PiB)^9^ and high-resolution volumetric magnetic resonance imaging (MRI) data.^2^, ^6^ PiB studies have confirmed what was predicted by earlier postmortem studies,^13^–^15^ that a substantial fraction (25–50%) of CN older individuals exhibit Aβ deposition.^16^–^21^ While still in an early phase, PET studies of Aβ deposition in these otherwise normal individuals suggest evidence of early brain dysfunction including disrupted default network functional connectivity,^19^, ^21^ aberrant default network activity during memory encoding,^18^ and even subtle cognitive impairment^22^, ^23^ that is offset by cognitive reserve.^23^ Here we relate the presence and pattern of Aβ-related atrophy observed in AD patients to the pattern seen in CN older individuals.

Atrophy can be quantified by automated measurement of brain MRI images, which yields estimated thickness measures of anatomically parcellated cortical regions as well as subcortical volumes.^24^–^27^ Such measurements have revealed a characteristic pattern of cortical thickness reductions and subcortical volume loss in clinically diagnosed AD patients.^25^, ^27^–^29^ The AD-like pattern of atrophy has also been reported in presymptomatic autosomal dominant AD,^28^ and in those with mild cognitive impairment who go on to develop the clinical diagnosis of AD.^30^ More recently, Desikan and colleagues^11^ identified a pattern of atrophy in the supramarginal cortex, entorhinal cortex, and hippocampus with which mild cognitive impairment (MCI) and AD could be distinguished from normal aging, and Davatsikos and colleagues^31^ identified a similar pattern of volume loss that related to cognitive decline among MCI as well as normal control subjects.^32^ However, these studies used control groups of older individuals in which amyloid was likely present, but the impact was not assessed. Studies directly relating structural data to Aβ deposition in CN subjects have yielded inconsistent results; while some have reported reduced hippocampal volume^33^, ^34^ or cortical thickness^29^, ^34^ in CN subjects with greater Aβ deposition, others have found this only among Aβ-positive CN^35^ or in normal individuals with subjective cognitive impairment.^36^ Similarly, the impact of age on amyloid and atrophy has not been consistently controlled. We sought to relate both hippocampal volume and cortical thickness reductions to a continuous measure of Aβ deposition adjusting for age in a large sample of both Aβ-positive and Aβ-negative CN subjects and in AD patients.

We first determined the pattern of cortical thickness reductions and hippocampal volume loss in mild AD patients compared to CN subjects, and then investigated whether a similar pattern of Aβ-associated volume loss was present in CN subjects. We also investigated the age-dependence of Aβ deposition and of Aβ-associated thickness reductions in both CN and AD, and quantified the extent and anatomic specificity of Aβ-related volume loss within each group. We hypothesized that Aβ deposition would be associated with local cortical thickness reductions in regions associated with the default network^37^ at early stages of the pathophysiological process, prior to cognitive impairment.

## Patients and Methods

### Subjects

Participants were recruited from ongoing longitudinal studies in aging and during screening for dementia clinical trials at the Massachusetts General and Brigham and Women's Hospitals, and from several local referring tertiary memory clinics (S.S., G.M., and D.M.). All participants were studied using protocols and informed consent procedures approved by the Partners Human Research Committee. All subjects underwent at least 1 comprehensive medical and psychiatric interview, as well as a neurological evaluation, to rule out any major medical or neurological disorders that might contribute to cognitive dysfunction. None of the participants had any notable medical or neurological illness, and none had a history of alcoholism, drug abuse, or head trauma, or a family history of autosomal dominant AD. None were clinically depressed (Geriatric Depression Scale <11; Yesavage and colleagues^38^**)** or had other psychiatric illnesses. Each participant was scored on the Mini-Mental State Examination (MMSE),^39^ and also underwent a standard battery of neuropsychological (NP) tests, as reported.^23^ The mean (standard deviation [SD]) time between PET imaging and testing was 0.90 (1.9) months.

Subjects were classified into 2 groups, CN (n = 87) and AD (n = 32). All CN subjects had a Clinical Dementia Rating (CDR) score of 0,^40^ MMSE > 27, and performance within 1.5 SD on age-and-education–adjusted norms on cognitive testing as detailed.^23^ AD subjects were CDR = 1 and satisfied criteria for a clinical diagnosis of probable AD according to National Institute of Neurological and Communication Disorders and Stroke/Alzheimer's Disease and Related Disorders Association criteria.^41^ Of the 87 CN subjects, 60 had apolipoprotein E (APOE) genotype data available: 47 were classified as ε4-negative (no ε4-alleles) and 13 as ε4-positive (one or two ε4-alleles).

### PET Acquisition and Processing

Carbon-11 PiB PET was acquired and processed as described, using the distribution volume ratio (DVR) with cerebellar cortex as reference tissue.^18^, ^42^ Detailed PET methods are discussed in the Supporting Information.

### MR Acquisition and Freesurfer Processing

High-resolution MRI images were acquired using magnetization-prepared rapid gradient-echo (MP RAGE) and processed with Freesurfer (FS) to measure cortical thickness and hippocampal volume, as described.^18^, ^19^ The FS-generated cortical parcellation defines a large precuneus region of interest (ROI) that overlaps areas of the posterior cingulate, including Brodmann area 23 and 31 along the posterior midline^43^; we therefore denoted this cortical ROI as the posterior cingulate/precuneus (PCC) in the following. Further details of FS processing are in the Supporting Information.

### Choice of Proxy Region for Aβ Deposition

As proxy ROI for Aβ deposition, we chose the PCC because it is a highly vulnerable, common site of early involvement. In addition, however, we evaluated 9 other ROIs that are also vulnerable to Aβ deposition to determine whether Aβ-associated volume/thickness changes differed when the proxy measure of PiB retention was from these alternative ROIs: rostral anterior cingulate, medial orbitofrontal, rostral middle frontal, caudal anterior cingulate, precuneus, superior frontal, pars opercularis, caudal middle frontal, inferior parietal, lateral orbitofrontal, and global.

### Dichotomization of PiB Data

Like earlier studies,^2^, ^18^, ^19^, ^23^, ^34^ we chose a threshold of amyloid positivity that is somewhat arbitrary, since a rigorous definition will likely require longitudinal follow-up. As in a previous report,^18^ we split the CN group based on partial-volume–corrected (PVC) PCC PiB retention: subjects with PCC DVR > 1.60 were classified as CN+ (PiB-positive CN), and those with DVR ≤ 1.60 as CN−. This is a conservative threshold and classifies fewer CN as PiB+ relative to other criteria (eg, Hedden and colleagues^19^). As described below, all analyses were also performed without the use of a threshold.

### Statistical Analyses

We evaluated the relation of hippocampal volume and cortical thickness to PiB primarily by treating PiB DVR as a continuous variable. Regression models were used to examine the relationship of hippocampal volume and cortical thickness to Aβ burden in CN and AD groups; regression coefficients for all models were estimated by ordinary least squares. At each cortical vertex thickness was taken as the dependent variable, and PVC PCC PiB DVR and age were taken as independent variables. Clusters of vertices with thickness-DVR regression coefficient *p*-values exceeding a predetermined threshold (*p* = 0.05) were identified, and cluster-wise statistical significances were calculated via 5000 instances of a Monte Carlo simulation, based on the noise distribution of the baseline analysis.^44^ We evaluated the relationship of hippocampal volume to PiB retention using similar regression models, with gender added as a covariate.^11^, ^45^–^47^ Hippocampal volumes (sum of volumes in left and right hemisphere) were covariance adjusted for total intracranial volume as measured by estimated total intracranial volume (eTIV) over the full sample prior to inclusion in the regression equation as the dependent variable.^48^

Parallel analyses were performed with regional average cortical thicknesses (average of left and right hemisphere thicknesses) from a set of anatomically defined cortical ROI: global (average over all cortical ROI), inferior parietal, PCC, parahippocampal, and entorhinal.^26^ ROI thickness was taken as the dependent variable, and PiB DVR and age as independent variables.

A hypothetical model of the relationship of cortical thickness and PiB retention was assessed under the assumption that both followed sigmoid curves, parameterized by a common time-like parameter (Supporting Information).

The age dependence of cortical thickness or hippocampal volume was investigated by regressing thickness on age, or volume on age and gender, in both the CN and AD diagnostic groups. Volume or thickness contrasts between diagnostic groups (CN and AD, or CN− and CN+) were assessed by analysis of covariance (ANCOVA) implemented as a general linear model, with age and gender covariates for volume, or age covariate for thickness.

In order to test whether age differences of cortical thickness or PiB uptake depended on APOE carrier status in the CN group, ε4 status (positive if one or two ε4-alleles, negative otherwise) was added to the model as a factor and allowed to interact with the regression term. Similarly, the differential effect of APOE status on the relationship of cortical thickness and PiB uptake in the CN group was assessed by including carrier status as a factor interacting with the thickness-PiB regression term.

The capacity of regional cortical average thickness to discriminate between the CN− and CN+ groups was assessed by logistic regression followed by receiving operating characteristic (ROC) curve analysis. Group membership probabilities predicted by the logistic regression model with thickness and age regressors were used to construct a ROC curve, and the area under the curve (AUC) and its statistical significance were computed (Wilcoxon rank-sum test with continuity correction).

## Results

### Subject Characteristics

The AD and CN groups differed in MMSE scores and PCC PiB retention, but not in age, gender, or education (Table 1). Hippocampal volumes were slightly greater in men compared to women even after residualization by intracranial volume (data not shown). Gender was therefore included as a factor in regression models involving hippocampal volume. Gender effects were not detected in PiB or cortical thickness data.

**TABLE 1:** Demographics

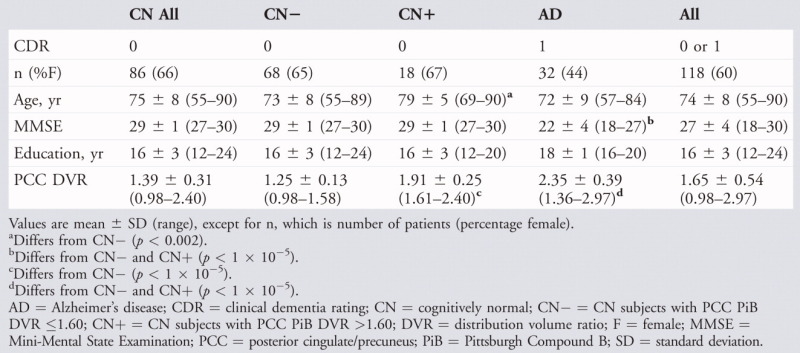

### Thickness/Volume Contrasts in AD vs CN

Reduced temporoparietal cortical thickness, controlling for age, was seen in AD compared to CN (Supporting Information Fig S1). Thickness decreases in AD relative to CN ranged up to 0.40mm (first/second/third quartiles = 0.20/0.26/0.30mm for vertices in the PCC). The anatomic pattern included posterior cingulate extending into the precuneus; inferior and superior parietal lobules; superior, middle, and inferior temporal; fusiform; entorhinal; parahippocampal, perirolandic, and posterior prefrontal regions. Anterior and medial prefrontal regions were less involved (Supporting Information Fig S1). In ROI contrasts, AD subjects had lower hippocampal volume (*p* < 1 × 10^−5^) and decreased entorhinal, parahippocampal, PCC, inferior parietal, and global thickness (*p* < 1 × 10^−5^), compared to the CN group (Supporting Information Fig S2).

### Aβ-Associated Cortical Thickness/Volume Reductions in AD and CN

#### Continuous Aβ (Vertex-Level Analyses)

**FIGURE 1:**
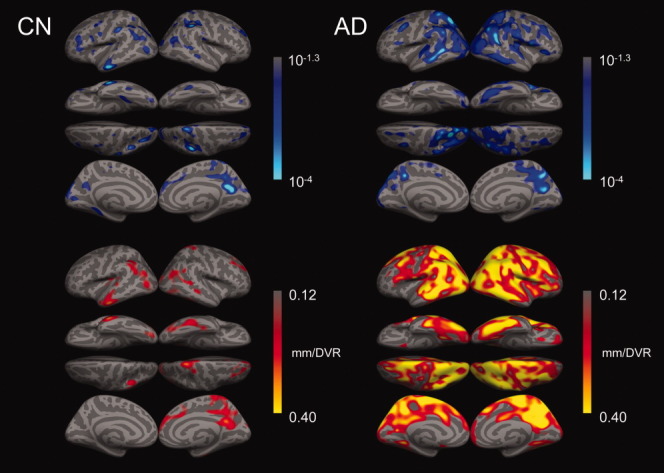
Aβ-associated reduction in cortical thickness in CN subjects and AD patients. Regression coefficients expressing reduction in thickness at each vertex per unit increase in PCC DVR controlling for age (bottom row), and corresponding statistical significance as *p* value (top row) in CN or AD groups (left or right column, respectively). Only clusters of 3000 or more contiguous vertices with regression coefficients exceeding 0.12mm/DVR are shown on the bottom row of surfaces.

Treating PCC PiB retention as a continuous measure and controlling for age, Aβ-associated cortical thickness reductions in both AD and CN subjects were seen in the posterior cingulate, extending into the precuneus, inferior parietal lobule, superior parietal, lateral temporal, and lateral prefrontal (Fig 1). In contrast to the AD vs. CN thickness contrast maps in Supporting Information Figure S1, significant medial temporal cortical Aβ-associated thickness reductions were not observed. No regions exhibited significant cortical thickness increases with increasing PiB retention.

In the CN group, cluster-wise statistical significance of vertex-level regression coefficients was assessed by Monte Carlo simulation to correct for capitalization on multiple comparisons. We identified 7 clusters of vertices as exhibiting significant thickness reductions with increasing Aβ at *p* < 0.05 (corrected): right posterior cingulate/precuneus, left inferior parietal, left and right rostral middle frontal, left and right supramarginal, and right superior temporal (Table 2; Supporting Information Fig S3). There were no areas of significant interaction between APOE carrier status and age-adjusted Aβ-associated thickness variation in the CN group (n = 60; data not shown), indicating that thickness-PiB regression slopes did not differ according to carrier status. To confirm that the observed significant inverse relation of Aβ and thickness was not an artifact of the PET partial volume correction, we substituted non-PVC PiB DVR for PVC DVR in the vertex-based regressions. Statistical significance of coefficients in the non-PVC analyses were lower in the CN group, as expected due to contraction of PET DVR ranges, but the pattern of the effects did not change (Supporting Information Fig S4).

**TABLE 2:** Reduction of Vertex Cortical Thickness with Increasing PCC PiB Retention, Controlling for Age in CN Subjects

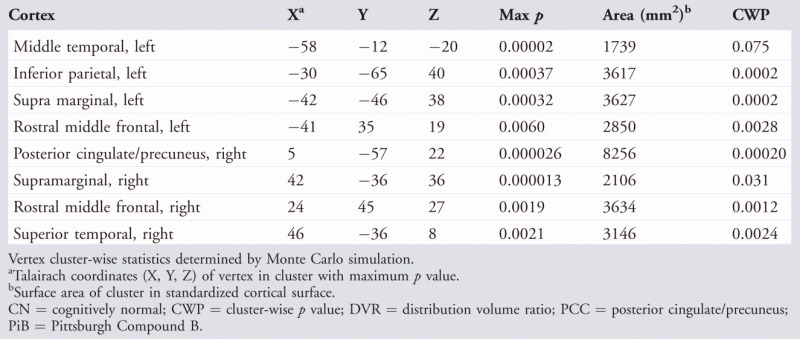

#### Continuous Aβ (ROI-Level Analyses)

**FIGURE 2:**
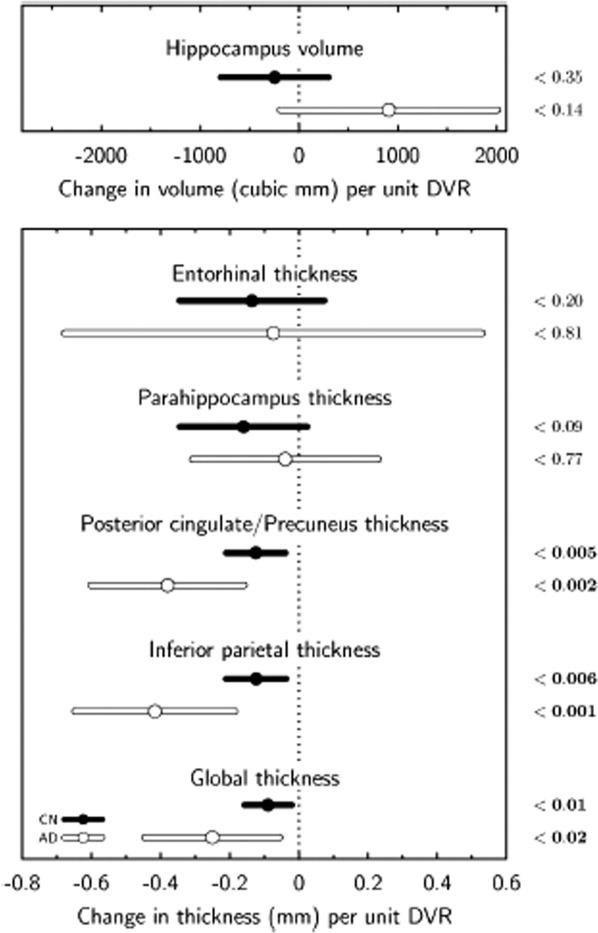
Aβ-associated hippocampal volume and regional thickness changes in CN and AD groups. Regression coefficients expressing change in hippocampal volume or regional average thickness per unit increase in PCC DVR controlling for age (age and gender for hippocampal volume), and corresponding 95% confidence intervals and statistical significances (right).

Data in ROI were expressed as age-adjusted structural change per unit change in PiB retention for hippocampal volume (mm^3^/DVR) and cortical thickness (mm/DVR) (Fig 2). Significant Aβ-associated cortical thickness reduction (significant negative regression coefficient expressing change in thickness per unit increase in DVR) was confirmed in PCC, inferior parietal, and global ROI, but entorhinal and parahippocampal thickness and hippocampal volume variations with Aβ were not statistically significant.

#### CN Group Dichotomized into CN−/CN+ by Aβ Level

The vertex-level contrast of CN− vs CN+ groups revealed age-adjusted thickness reduction in the CN+ group prominently in posterior cingulate/precuneus, lateral parietal, and prefrontal cortices (Supporting Information Fig S5). Thickness decreases in CN+ relative to CN− ranged up to 0.19mm (first/second/third quartiles = 0.034/0.064/0.090mm for vertices in the PCC). In ROI contrasts of CN− vs CN+ groups, lower ROI average thicknesses were observed in the CN+ group but the differences did not reach statistical significance possibly because of lower sensitivity (Supporting Information Fig S2). Using PCC thickness to discriminate CN− and CN+ subjects yielded a statistically significant (*p* < 0.05) logistic regression model in which a 0.1mm decrease was associated with an odds ratio of 1.60. The corresponding AUC = 0.70 (*p* < 0.01), holding age constant at its grand mean. Other regions examined (parietal, frontal, or global average) did not achieve a similar discriminative efficiency.

### Thickness-Aβ Sigmoid Modeling

**FIGURE 3:**
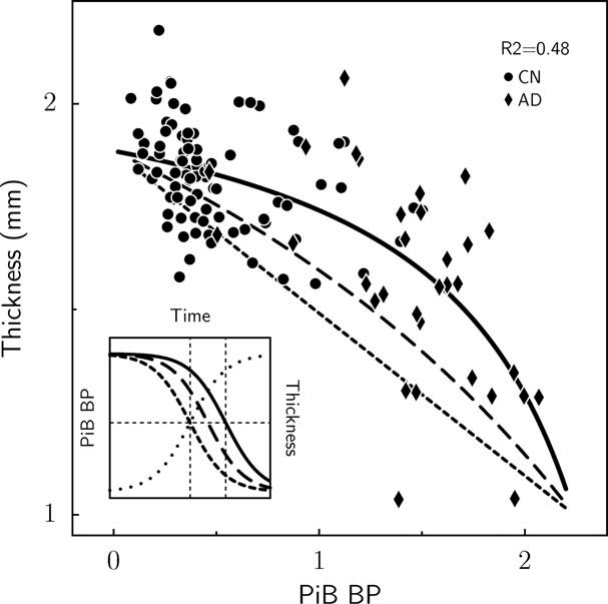
Modeling of PCC thickness as a function of PiB retention in CN and AD groups. Least squares fit *(solid curve)* of thickness-PiB functional relationship based on sigmoid time courses, with the maximum rate of thickness decline later in time than the maximum rate of PiB increase; compare *solid* (thickness) and *dotted* (PiB) sigmoids *(inset graph)*. *Dashed curves* correspond to shorter time lags, *long-dashed curves* correspond to one-half the best-fit time lag, and *short-dashed curves* correspond to no lag. The *inset* shows the underlying sigmoid time courses for PiB *(dotted)* and thickness at the 2 time lags. As the time lag between thickness and PiB increases, the curvature of the thickness-PiB curve increases. Binding potential (which is equal to DVR − 1) was used as the PiB measure in the modeling since we assumed that PiB BP asymptotes to zero prior to disease onset.

While Aβ-associated thinning was observed in CN subjects as described above: (1) thinning was more anatomically extensive in the AD group; and (2) significantly more thinning per unit DVR was observed in the AD group (eg, 0.4mm/DVR in medial and lateral parietal areas) than in the CN group (see Fig 1). A vertex-level assessment of the difference revealed a significant interaction of the thickness-vs-Aβ coefficient and clinical status factor (CN vs AD) in posterior midline and inferior parietal regions (*p* < 1 × 10^−4^; data not shown). We related these observations to candidate time courses along the hypothetical CN–AD trajectory (see Supporting Information), in which the data were evaluated using sigmoid models to relate PCC cortical thickness and PiB retention. Assuming sigmoid time functions for PiB increase and loss of cortical thickness, both with the same rate-of-change achieved at the midpoint of the S-shaped portion of the curves, we calculated how far apart in time these midpoints would have to be in order to achieve the best fit to our data. In fitting this model to the age-adjusted CN and AD group data (Fig 3; *r*^2^ = 0.48; *p* < 1 × 10^−4^) we calculated this time lag parameter (see Supplemental Material) to be equal to 0.35 times the amyloid saturation time (the time to go from zero to maximum amyloid). For example, if a 10-year amyloid saturation time were hypothesized, the time lag between the rapid phases of PiB increase and cortical thinning would be 3.5 years.

### Age Dependence of Thickness/Volume and of PiB Retention in CN and AD

Vertex-level analyses revealed that greater age was associated with reduced thickness among CN subjects in perirolandic, lateral and inferior temporal, superior parietal, posterior cingulate, and precuneus cortices (*p* < 0.0001, uncorrected for multiple comparisons; see Supporting Information Fig S6). The vertex-based findings were confirmed in cortical ROI, where age-related reductions were also seen in parahippocampal, inferior parietal and global cortical thickness, and marginally in entorhinal thickness in the CN group. Age-associated hippocampal volume reduction was also significant in the CN group. In the AD group, greater age was associated with decreased volume in hippocampus and reduced thickness prominently in parahippocampal cortex (Supporting Information Fig S6).

Analyses of PiB data derived either from vertices or from confirmatory ROI revealed that while higher PiB retention in precuneus/posterior cingulate, anterior cingulate, and prefrontal regions was associated with greater age in the CN group, the inverse relation was seen in the AD group (Supporting Information Fig S7). For example, age was associated with increased PCC ROI PiB retention in the CN group (*p* < 0.001), but with decreased PCC retention in the AD group (*p* < 0.005). This corresponded to a significant difference in the regression line slopes between the 2 groups (*p* < 1 × 10^−4^). Prominently reduced Aβ deposition as a function of age was observed in medial occipital regions in AD (Supporting Information Fig S7). When non-PVC data were used, the age associations were similar but less robust possibly due to the narrower ranges of uncorrected DVR (data not shown).

There were no areas of significant interaction between APOE carrier status and variation with age of cortical thickness or PiB retention in the CN group (n = 60; data not shown).

### Differential Impact of Age and Aβ Deposition on Hippocampal Volume and Cortical Thickness

Maps of cortical thickness for the age regression coefficient were computed with the Aβ deposition term also included in the model. These were nearly identical to the maps of the simple age-dependence (Supporting Information Fig S6), indicating that the age was inversely related to thickness independent of Aβ deposition (data not shown).

Using ROI data we evaluated a regression model that included age, gender, and PCC PiB retention as predictors of PCC ROI thickness or hippocampal ROI volume. PCC thickness was independently associated with both age (*p* < 1 × 10^−3^) and Aβ deposition (*p* < 0.004). In contrast, hippocampal volume was associated with age (*p* < 1 × 10^−5^) but not with Aβ deposition (*p* = 0.38). Gender was not related to either hippocampal volume (preadjusted for eTIV; *p* < 0.07) or PCC thickness (*p* < 0.45). When added to the models, the age-by-Aβ interaction term was not significant in either case. Thus, covarying Aβ deposition, greater age was associated with reduced hippocampal volume and PCC thickness; however, covarying age, Aβ deposition was associated with reduced PCC thickness but not with reduced hippocampal volume.

### Choice of Proxy for PiB Retention

We tested the hypothesis that results using alternative, non-PCC regions would differ from those in which the PCC was used as the proxy for Aβ deposition. We found that using as proxy the rostral anterior cingulate, rostral middle frontal, or inferior parietal cortices all yielded similar patterns of Aβ-associated cortical thinning, which was not surprising given the high correlation of PiB retention in these regions with PiB retention in the PCC (Pearson correlations ranged from 0.80 to 0.89) In contrast, hippocampus PiB retention was not significantly related to thinning in any cortical region (data not shown).

## Discussion

The major finding of this study is that significant Aβ-associated cortical thinning occurs among CN older individuals in a pattern consistent with early AD. While this finding supports the possibility that Aβ deposition in normal individuals represents preclinical AD, direct observation with longitudinal data will be required to evaluate the strength and timing of this link. Our data suggest that Aβ-associated neurodegeneration manifests as cortical thinning in regions vulnerable to early Aβ deposition, including association cortices along the posterior medial wall and lateral parietal cortex. In particular, we observed thinning in the inferior parietal lobule and the posterior cingulate extending into the precuneus, which are regions that form nodes of a large-scale cortical system known as the default network.^37^, ^49^, ^50^ This system has been implicated in both memory-related function and in amyloid-related and AD-related memory dysfunction.^18^, ^37^, ^50^–^53^ Our findings are consistent with a pathophysiologic link between Aβ deposition and neurodegeneration in this network, which may anticipate memory failure and progression to clinical dementia.^18^, ^19^, ^21^, ^37^, ^54^

Along the posterior midline, the posterior cingulate and retrosplenial cortex are anatomically connected to medial temporal structures, and we found that while the hippocampus and medial temporal lobe (MTL) cortices demonstrated significant age-associated atrophy, the association of MTL atrophy with Aβ deposition was variable in these asymptomatic older individuals. While our results are consistent with the hypothesis that MTL atrophy coincides with the emergence of manifest cognitive impairment,^2^, ^55^, ^56^ we did not observe a significant difference between MTL and cortical atrophy, and thus cannot order the relative timing of effects with the present data. While macroscopically-visible cortical atrophy is associated with dementia, it is not generally observed in nondemented individuals at postmortem,^57^ perhaps because of an inability to differentiate it from normal age-associated atrophy. Our data suggest that the Aβ deposition commonly detected in normal older individuals is associated with subtle posterior cingulate and parietal neurodegeneration that occurs prior to, and may be a harbinger of, clinically significant impairment.^29^ It is possible that further investigation will reveal evidence of subtle cognitive alterations related to cortical thinning even within CN individuals, particularly when the level of cognitive reserve is considered.^23^

More broadly, our findings should be considered in the context of a putative sequence of events in AD pathology that can be observed with biomarkers. Using a largely biphasic model of disease sequence, Aβ deposition has been hypothesized to occur early in the sequence of AD pathology and to be followed later by neurodegeneration, which is then related to the symptomatic phases of the disease, cognitive decline and dementia.^58^–^60^ Our present findings and those of earlier studies^29^, ^33^, ^34^ that suggest PiB retention is correlated with cortical thinning in normal individuals raise the possibility that the hypothesized lag period between Aβ deposition and neurodegeneration may be shorter than previously thought. A precise mechanism by which Aβ deposition could be linked to neurodegeneration has not been firmly established. It is possible that toxic effects of Aβ oligomeric assemblies that surround fibrillar forms could be exerted locally early in the process and result directly in synapse and cell loss.^61^, ^62^ Such a mechanism entails a direct relationship between the presence of Aβ and neurodegeneration, which could potentially be observed with sensitive biomarkers. However, while the sensitivity of Aβ imaging may be improved in the future and permit better detection, individuals with predominantly prefibrillar or polymorphic forms of Aβ that are refractory to PiB would not be detectable with PiB or likely with other thioflavin or Congo-red derivatives.^63^–^65^

We found that Aβ and PCC thickness were more strongly correlated in AD than in CN (see Fig 1) and we evaluated these data according to a recently proposed model^59^ in which Aβ deposition and cortical thickness follow sigmoid-shaped curves in time. We simultaneously fit sigmoid models to PCC PiB and age-adjusted thickness data for the combined CN and AD groups, and determined the temporal lag between the dynamic phases of amyloid accumulation and cortical thinning to be approximately 35% of the total time required for amyloid to rise from its baseline to maximum. It should be emphasized that the model in its particularities as presented here is tentative, and should be considered as a schematic rather than definitive treatment of the problem. Such modeling will remain speculative as to accurate parameters of the underlying sigmoid curves until longitudinal data are available.

Whereas AD neurodegeneration is well established to occur prominently in the MTL^55^, ^56^, ^66^ and to be correlated with neurofibrillary pathology,^67^ our findings are consistent with emerging evidence that thinning in posterior association cortices is also a prominent feature of MCI and AD.^11^, ^28^, ^29^, ^37^, ^48^, ^68^ While the measured amount of age-adjusted thickness reduction per unit of PiB retention (DVR) was approximately the same in the posterior cingulate/precuneus and in MTL structures, the standard errors were larger in the MTL (see Fig 1) and the regression coefficients did not reach significance. Future work with a larger data sample will be required to order the relative emergence of effects between posterior cingulate and MTL structures. Neurofibrillary tangle pathology may partially explain this observation of greater variability in the MTL, since it is common in MTL but rarely widespread in cortex of CN subjects.^57^, ^58^, ^67^, ^69^ Our data are not consistent with previous observations that Aβ deposition is only seen after significant neurofibrillary tangle deposition and MTL atrophy,^69^ but instead suggest that the pathologic sequence of events in preclinical AD is one in which Aβ deposition is related to neurodegeneration in posterior cingulate and distributed regions of association neocortex that occurs along with or possibly even prior to hippocampal and entorhinal neurodegeneration.

Previous reports of the relation of PiB retention and hippocampal volume in CN subjects have been inconsistent. Some reported an inverse relation (ie, decreased volume with increased PiB retention) in CN subjects,^33^, ^34^ while others found such a relation only among the Aβ-positive CN group^35^ or only in CN subjects with subjective cognitive impairment.^36^ These studies have differed in their treatment of the potentially confounding effect of age on both Aβ level and atrophy. We evaluated hippocampal volume, cortical thickness, and PiB retention for evidence of age-dependence and found evidence for an age effect in all domains. The age-dependence of volume/thickness across a broad age range has been previously reported,^70^ and although some investigators have not found a significant impact of age within a more restricted older age range,^29^ others have applied an age-adjustment to thickness/volume data.^11^ The strong age-dependence of Aβ deposition we observed in CN subjects is consistent with neuropathological studies that inferred from cross-sectional data that Aβ gradually accumulates with age.^68^ Several PiB studies^9^, ^16^, ^71^ did not report evidence of a significant relationship with age, perhaps due to the small sample sizes with limited age ranges, although a recent study did demonstrate an age association.^72^ Morris and colleagues^73^ found that the age-dependence of PiB data was largely accounted for by the strong age association with PiB retention among carriers of the APOE4 allele,^34^ likely a reflection of the sample enrichment for younger CN subjects with positive family histories. We did not find an interaction of the age-Aβ relationship with APOEe4 carrier status among the subset of subjects on whom genotypes were available (data not shown).

Interestingly, the age-dependence of Aβ deposition among CN subjects was reversed in AD patients, such that greater age was associated with lower levels of Aβ deposition. The reversal of the Aβ-age coefficient in the AD group compared to the CN group could be due to a survivor effect, such that older subjects with greater amounts of Aβ were too impaired (eg, MMSE < 18) to have been included in our study. Other potential factors, including an age-related change in PiB binding sites or affinity or a change in the production or clearance of Aβ,^74^, ^75^ will require further study. Moreover, longitudinal observation over long intervals may be required to determine whether individual AD patients' levels of Aβ decline over time, which has not been observed in longitudinal PiB data that has spanned 1 to 2 years.^2^, ^5^, ^76^

In summary, our findings provide support for the hypothesis that Aβ is associated with local neurodegeneration in key nodes of a distributed network supporting memory processes, and that this process begins prior to clinically-evident cognitive impairment, but continues into the stage of clinical dementia. Longitudinal follow-up of these CN older individuals is ongoing to determine if the combination of Aβ burden and volumetric loss is predictive of incipient cognitive decline, and progression to AD dementia.
