# Supplementary material for: Amyloid-β Associated Cortical Thinning in Clinically Normal Elderly
Source: Ann Neurol. 2011 Mar 17;69(6):1032–42. doi: 10.1002/ana.22333 (PMC3117980; doi:10.1002/ana.22333)
Supplement: Supplementary file 1 [file ana0069-1032-SD1.doc]

Amyloid-beta Associated Cortical Thinning in Clinically Normal Elderly

Becker JA, Hedden T, Carmasin J, Maye J, Rentz D, Putcha D, Fischl B, Greve D, Marshall GA, Salloway S, Marks D, Buckner RL, Sperling RA, Johnson KA.

**SUPPORTING SECTION**

**Supporting Methods:**

**PET acquisition:**

Pittsburgh Compound B (PiB) was prepared at Massachusetts General Hospital and subjects underwent PiB PET as described previously^1-4^. Briefly, data were acquired using a Siemens/CTI (Knoxville, TN) ECAT HR+ scanner (3D mode; 63 image planes; 15.2cm axial field of view; 5.6mm transaxial resolution and 2.4mm slice interval; 69 frames: 12x15 seconds, 57x60 seconds). After a transmission scan, 8.5 to 15mCi 11C-PiB was injected as a bolus and followed immediately by a 60-minute dynamic acquisition. PET data were reconstructed and attenuation corrected, and each frame was evaluated to verify adequate count statistics and absence of head motion.

**MR image acquisition:**

High-resolution T1-weighted structural images were acquired with a Siemens Trio 3.0 T scanner (Siemens Medical Systems, Erlangen Germany) using one of two similar 3D Magnetization Prepared Rapid Acquisition Gradient Echo (MP-RAGE) sequences: (1) repetition time (TR) = 2530 msec, echo time (TE) = 3.45 msec, inversion time (TI) = 1100 msec, flip angle (FA) = 7°, field of view (FOV) = 256 mm, matrix 192 x 256, slice thickness = 1.33 mm, 128 sagittal slices. (2) TR = 2300, TE = 2.98, TI = 900, FA = 9, FOV = 256, matrix 192 x 256, slice thickness = 1.0 mm, 192 sagittal slices, GRAPPA 2x acceleration. In order to assess the impact of variations in the voxel dimensions of MP-RAGE data in the sample, a factor representing MR acquisition type (with two levels: isotropic 1 mm^3 and anisotropic with slice thickness >1 mm) was added to all regression analyses. It was found that explicitly accounting for variation in MR acquisition parameters in this fashion did not substantively change the reported results.

**MR image processing:**

The Freesurfer (FS) software package was used to determine cortical (i.e., grey-matter ribbon) thickness and hippocampal volume^5, 6^. Briefly, FS derives grey/white-matter and CSF/gray-matter ("pial") surfaces from each subject's MPRAGE data set, with each surface having the same number of vertices. The cortical thickness associated with each pair of vertices is defined as the average of two distances: the distance of the white-matter surface vertex to the nearest point in the pial surface and the corresponding distance from the pial vertex to the white-matter surface. Inter-subject comparisons of cortical thickness measurements were performed on a standardized surface provided by FS, into which each subject’s cortical surface was non-linearly warped as part of FS processing. Cortical thickness data were then smoothed at FWHM 15mm on the surface in standardized space prior to further analysis. This cortical thickness measurement technique has been validated via histological^7^ as well as manual measurements^8, 9^. FS also yielded for each subject’s MR data set a probabilistic, atlas-based parcellation of the cortical surface into a set of cortical ROIs; average thickness associated with each cortical ROI was computed as the mean cortical thickness over all vertices in the ROI^6, 10^.

**PET image processing:**

The average of the first eight minutes of the dynamic PET PiB data and the structural MP-RAGE volume for each subject were brought into spatial correspondence using the SPM8 "coregister" tool^11^, which computed a six-parameter rigid body transformation relating the two acquisition spaces. The FS-defined cortical and subcortical ROIs from each subject’s MPRAGE were resampled into the native acquisition space of the PET using this linear co-registration transformation and average activities in each resampled ROI were computed in each frame of the dynamic PiB data set. The resulting regional time-activity curves (TAC) and the cerebellar-cortex reference region TAC were used to compute regional DVRs for each ROI using the Logan graphical method applied to data from 40 to 60 minutes after injection^2, 12-14^. Partial volume corrected (PVC) TACs were calculated using the two-component method of Meltzer^15^. FS-defined grey and white matter segments were combined to derive a binary brain image, which was convolved with the PSF of the PET camera (6.5 mm FWHM isotropic Gaussian) to yield the PV correction factor at each voxel of the native PET PiB volume. These PVC TACs were used to compute regional PVC DVRs as described above for uncorrected TACs.

Each subject’s PiB parametric DVR volume was also mapped onto the FS-derived cortical surface in native MR space by sampling at the midpoint of the grey-matter ribbon. The DVR at each vertex was PV corrected using the correction factor derived from the convolved binary brain mask sampled at the same grey-matter ribbon midpoint. Surface-mapped PVC PiB data was resampled onto a FS-defined cortical surface in standardized space to allow inter-subject comparisons, using the FS-computed nonlinear warping transformation relating the subject’s MR native cortical surface to the standardized surface space. Vertex-level PiB data were smoothed with a 15mm FWHM smoothing kernel prior to further calculation to reduce noise and compensate for potential registration error.

**Sigmoid modeling:**

The time courses of cortical thickness and PiB retention measured as binding potential (BP) were both assumed to be sigmoid-shaped and parameterized by a common time parameter, t:

BP(t) = BP0/(1+exp(-t/lambdaP))    and    T(t) = T0/(1+exp((t-t_T)/lambdaT))

where BP(t) and T(t) denote PiB binding potential and cortical average thickness (which are assumed to asymptote at zero and their respective maxima, BP0 and T0), lambdaP and lambdaT are time-scales characterizing the dynamics of BP and thickness, respectively, and t_T is the time lag of the thickness sigmoid relative to the PiB sigmoid. The PiB sigmoid was assumed to achieve its maximum rate of change at time t=0. A direct functional relationship T(BP) between thickness and PiB BP was derived by eliminating t between these two equations, and was characterized by four parameters: BP0, T0, the time-scale ratio lambdaP/lambdaT, and the lag parameter delta=t_T/lambdaP.  The PiB and thickness time-scales were assumed to be equal for simplicity, i.e., lambdaP = lambdaT.  The thickness-PiB function T(BP) was fit to age-adjusted thickness-PiB data for the combined CN and AD group by nonlinear least squares minimization (R function nls), varying the parameters BP0, T0 and delta. The resulting dimensionless time lag parameter delta, was converted to an estimate in years based on an independent estimate of the PiB time-scale lambdaP. Approximating the rise of the PiB sigmoid BP(t) from 0 to its maximum BP0 by the tangent at its midpoint BP(t=0), the time required for PiB to saturate -- the amyloid saturation time TPiB -- is TPiB = 4*lambdaP.  Then the time lag t_T = delta*lambdaP = (delta/4)*TPiB.

**References**

1. Mathis CA, Wang Y, Holt DP et al. Synthesis and evaluation of 11C-labeled 6-substituted 2-arylbenzothiazoles as amyloid imaging agents. J Med Chem. 2003;46:2740-2754

2. Gomperts SN, Rentz DM, Moran E et al. Imaging amyloid deposition in Lewy body diseases. Neurology. 2008;71:903-910

3. Hedden T, Van Dijk KR, Becker JA et al. Disruption of functional connectivity in clinically normal older adults harboring amyloid burden. J Neurosci. 2009;29:12686-12694

4. Sperling RA, Laviolette PS, O'Keefe K et al. Amyloid deposition is associated with impaired default network function in older persons without dementia. Neuron. 2009;63:178-188

5. Fischl B, Liu A, Dale AM. Automated manifold surgery: constructing geometrically accurate and topologically correct models of the human cerebral cortex. IEEE Trans Med Imaging. 2001;20:70-80

6. Desikan RS, Cabral HJ, Hess CP et al. Automated MRI measures identify individuals with mild cognitive impairment and Alzheimer's disease. Brain. 2009;132:2048-2057

7. Rosas HD, Liu AK, Hersch S et al. Regional and progressive thinning of the cortical ribbon in Huntington's disease. Neurology. 2002;58:695-701

8. Salat DH, Buckner RL, Snyder AZ et al. Thinning of the cerebral cortex in aging. Cereb Cortex. 2004;14:721-730

9. Dickerson BC, Bakkour A, Salat DH et al. The cortical signature of Alzheimer's disease: regionally specific cortical thinning relates to symptom severity in very mild to mild AD dementia and is detectable in asymptomatic amyloid-positive individuals. Cereb Cortex. 2009;19:497-510

10. Desikan RS, Segonne F, Fischl B et al. An automated labeling system for subdividing the human cerebral cortex on MRI scans into gyral based regions of interest. Neuroimage. 2006;31:968-980

11. Friston KJ, Holmes AP, Worsley JB et al. Statistical Parametric Maps in Functional Imaging: A General Linear Approach. Human Brain Mapping. 1995;2:189-210

12. Lopresti BJ, Klunk WE, Mathis CA et al. Simplified quantification of Pittsburgh Compound B amyloid imaging PET studies: a comparative analysis. J Nucl Med. 2005;46:1959-1972

13. Klunk WE, Mathis CA. The future of amyloid-beta imaging: a tale of radionuclides and tracer proliferation. Curr Opin Neurol. 2008;21:683-687

14. Logan J, Fowler JS, Volkow ND et al. Distribution volume ratios without blood sampling from graphical analysis of PET data. J Cereb Blood Flow Metab. 1996;16:834-840

15. Meltzer CC, Zubieta JK, Links JM et al. MR-based correction of brain PET measurements for heterogeneous gray matter radioactivity distribution. J Cereb Blood Flow Metab. 1996;16:650-658

**Supporting Figures:**

**
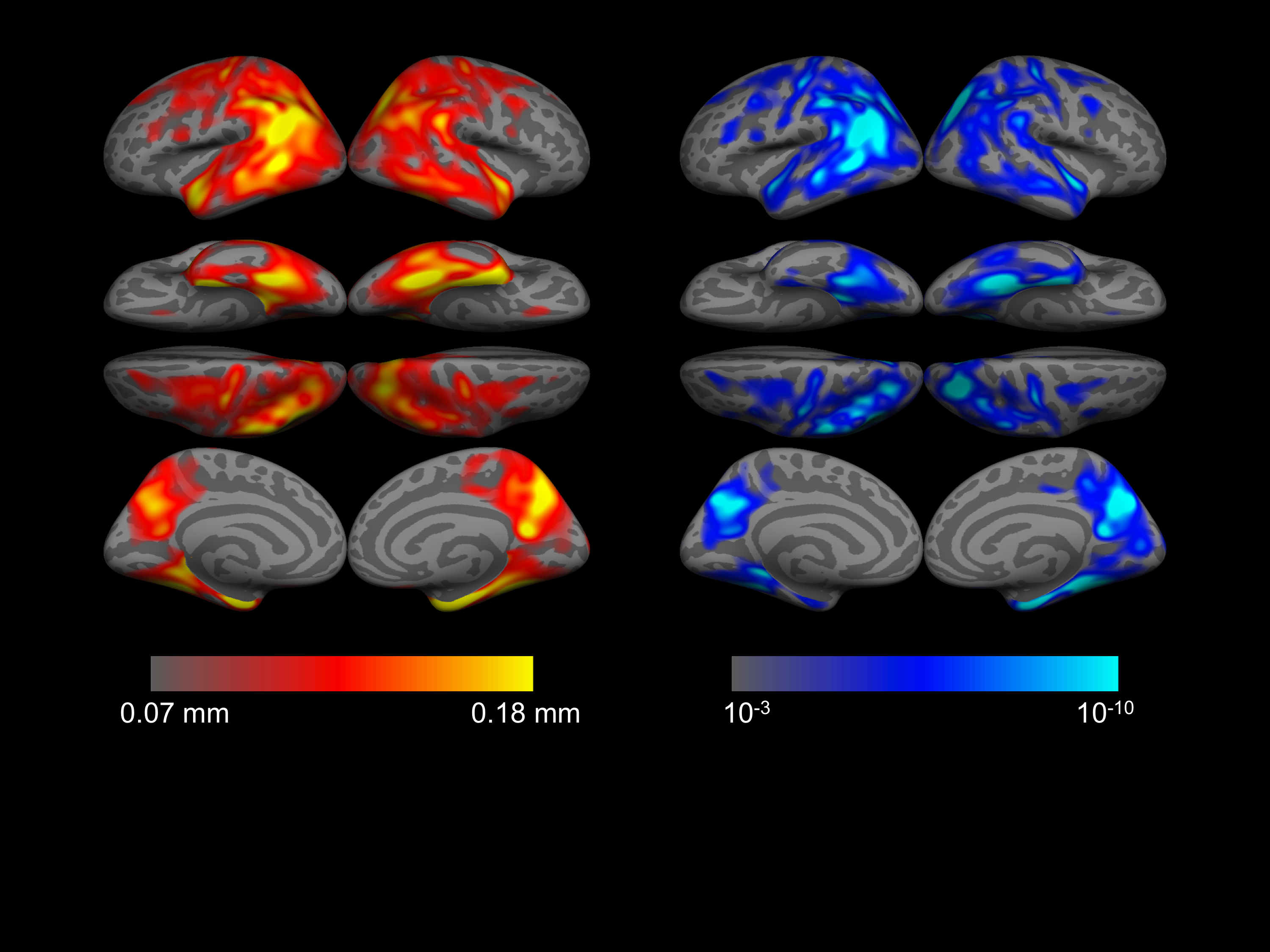
**

**Supporting Figure S1. Reduced cortical thickness in Alzheimer’s disease patients compared to clinically normal (CN) subjects.** Age-adjusted reduction in thickness at each vertex in mm in AD patients compared to CN subjects (left), with statistical significances (p-values) (right). Surfaces are shown in lateral (upper row), inferior and superior (middle), and medial (lower row) views, with dark grey regions representing sulci and light grey regions representing gyri.


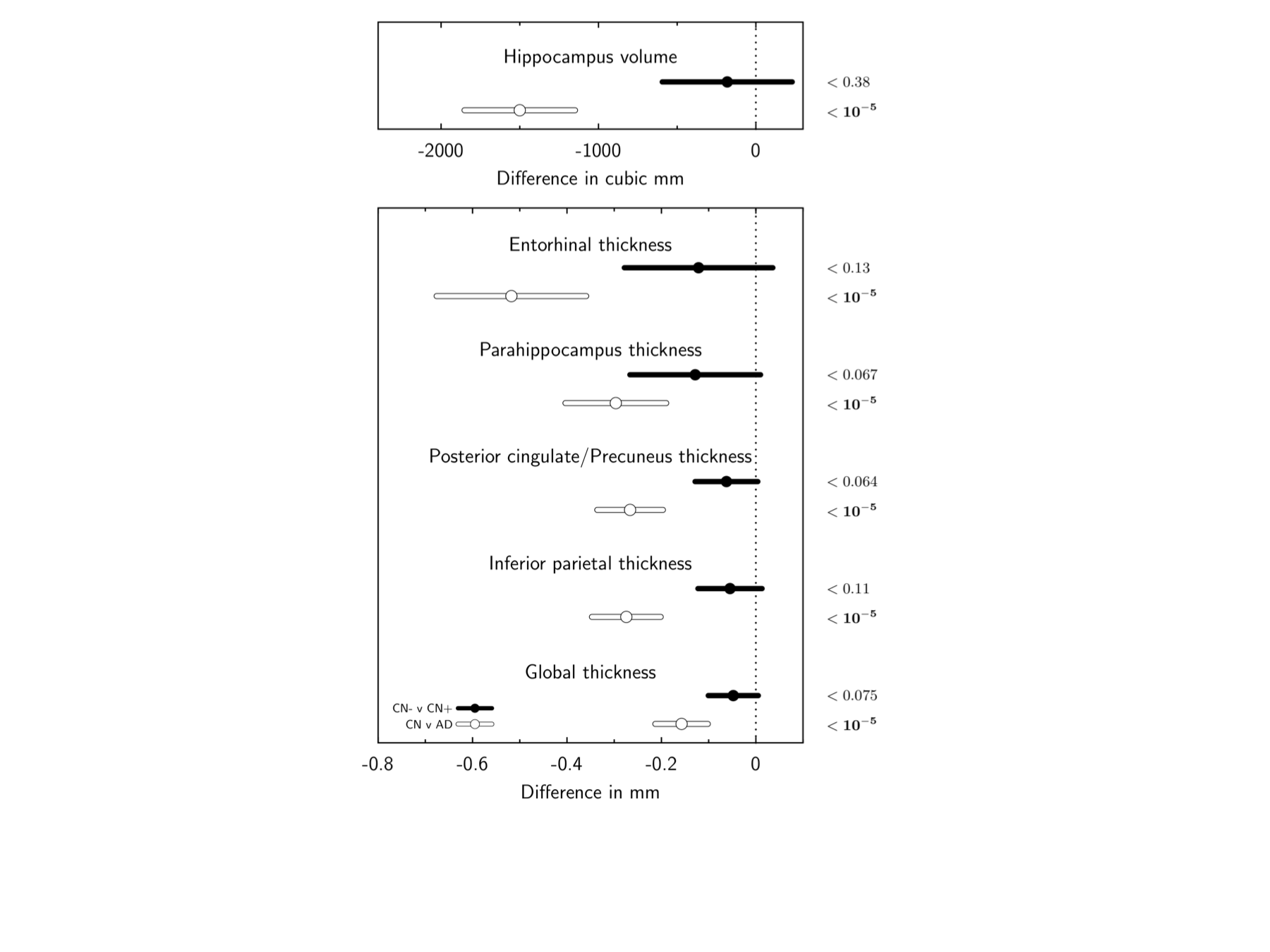
**Supporting Figure S2. Reduced hippocampal volume and cortical thickness in CN+ compared to CN- subjects and AD patients compared to CN subjects.** eTIV-adjusted hippocampal volume and average regional thickness differences (CN+ minus CN-), 95% confidence intervals and p-values, controlling for age (age and gender for hippocampal volume).


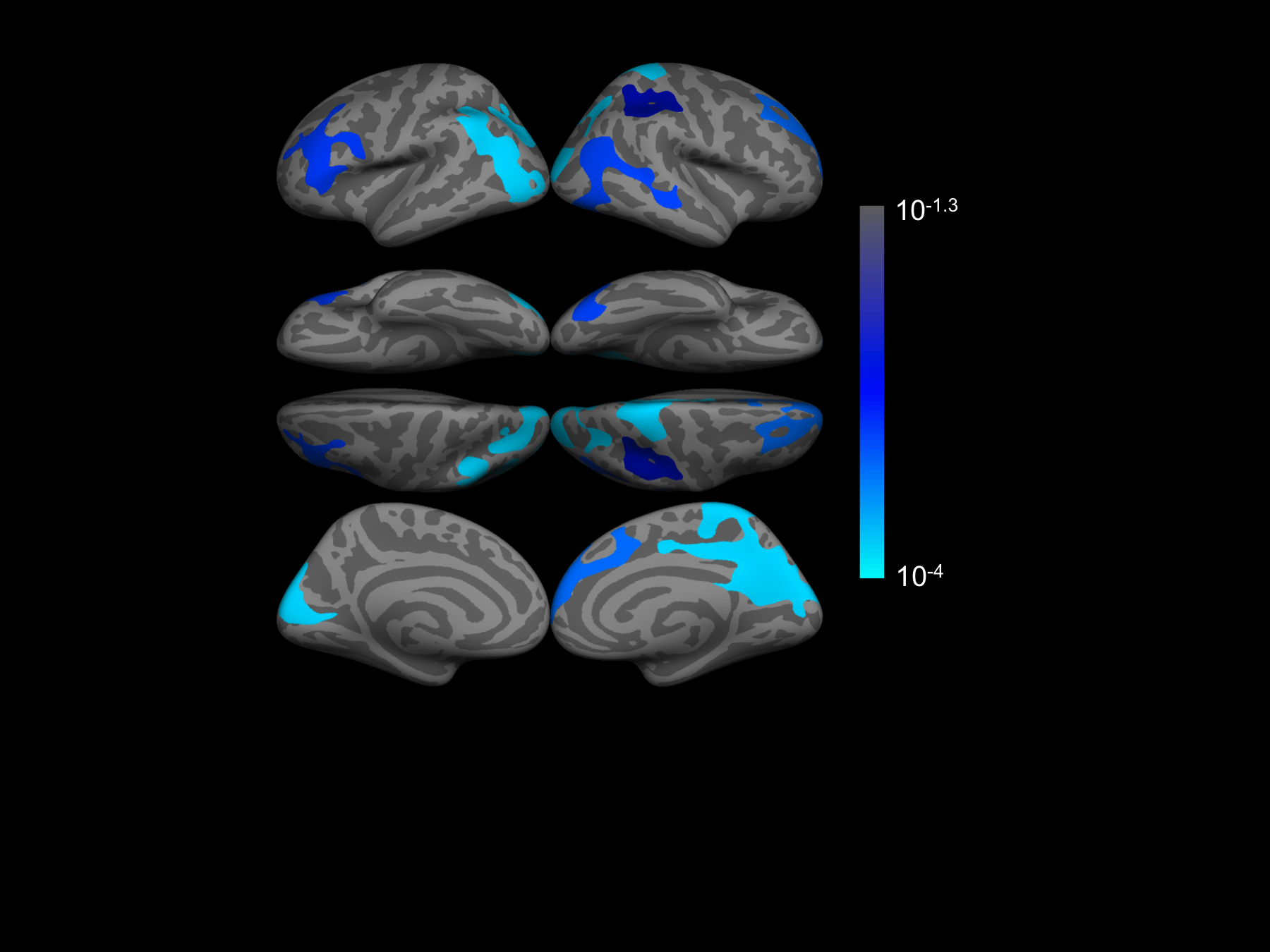


**Supporting Figure S3. Cluster-wise statistical significance of reduction in cortical thickness with increasing PCC PiB retention controlling for age in CN subjects.** Clusters of vertices with p<0.05 were identified and cluster-wise p-values computed by Monte Carlo simulation.


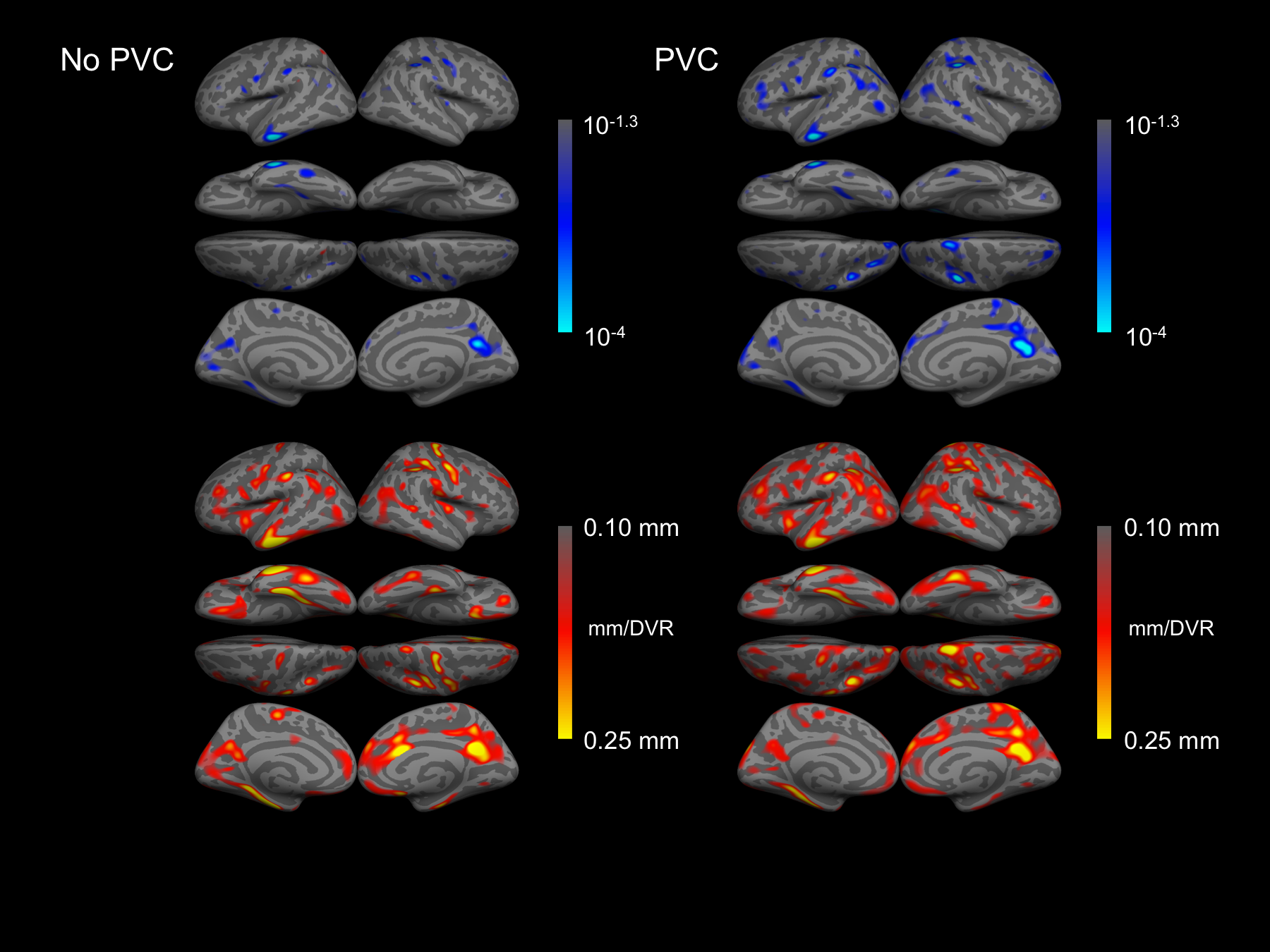


**Supporting Figure S4. The effect of partial-volume correction on thickness-PiB slopes in CN subjects.** Regression coefficients expressing reduction in thickness at each vertex per unit increase in PCC DVR controlling for age (bottom row), and corresponding statistical significance as p-value (top row) with PV correction (right column) or no such correction (left).

**
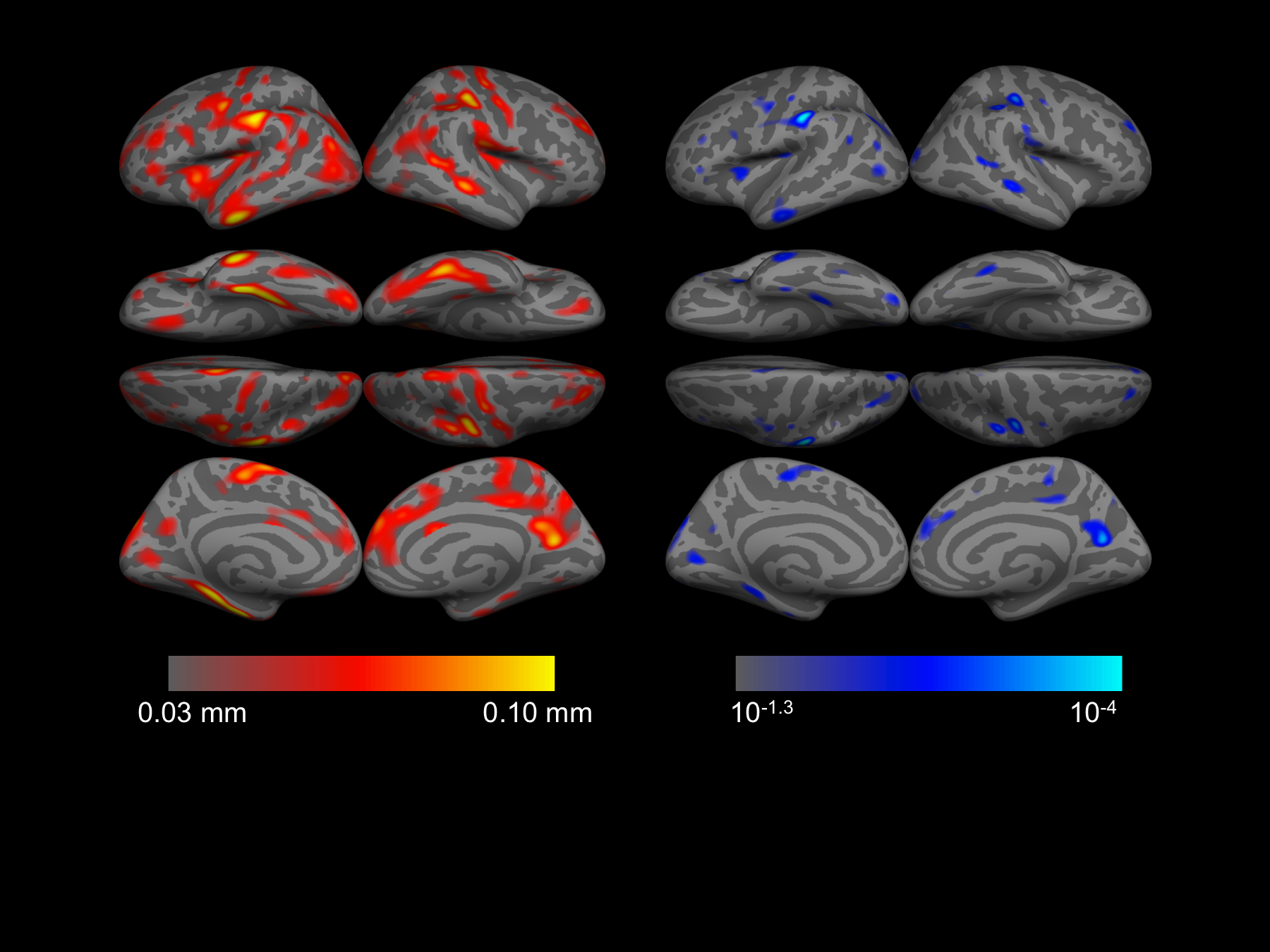
**

**Supporting Figure S5: Reduced cortical thickness in Aβ-positive (CN+) compared to Aβ-negative (CN-) clinically normal subjects.** Age-adjusted reduction in thickness at each vertex in mm (left), corresponding statistical significances (p-values) at right.

**
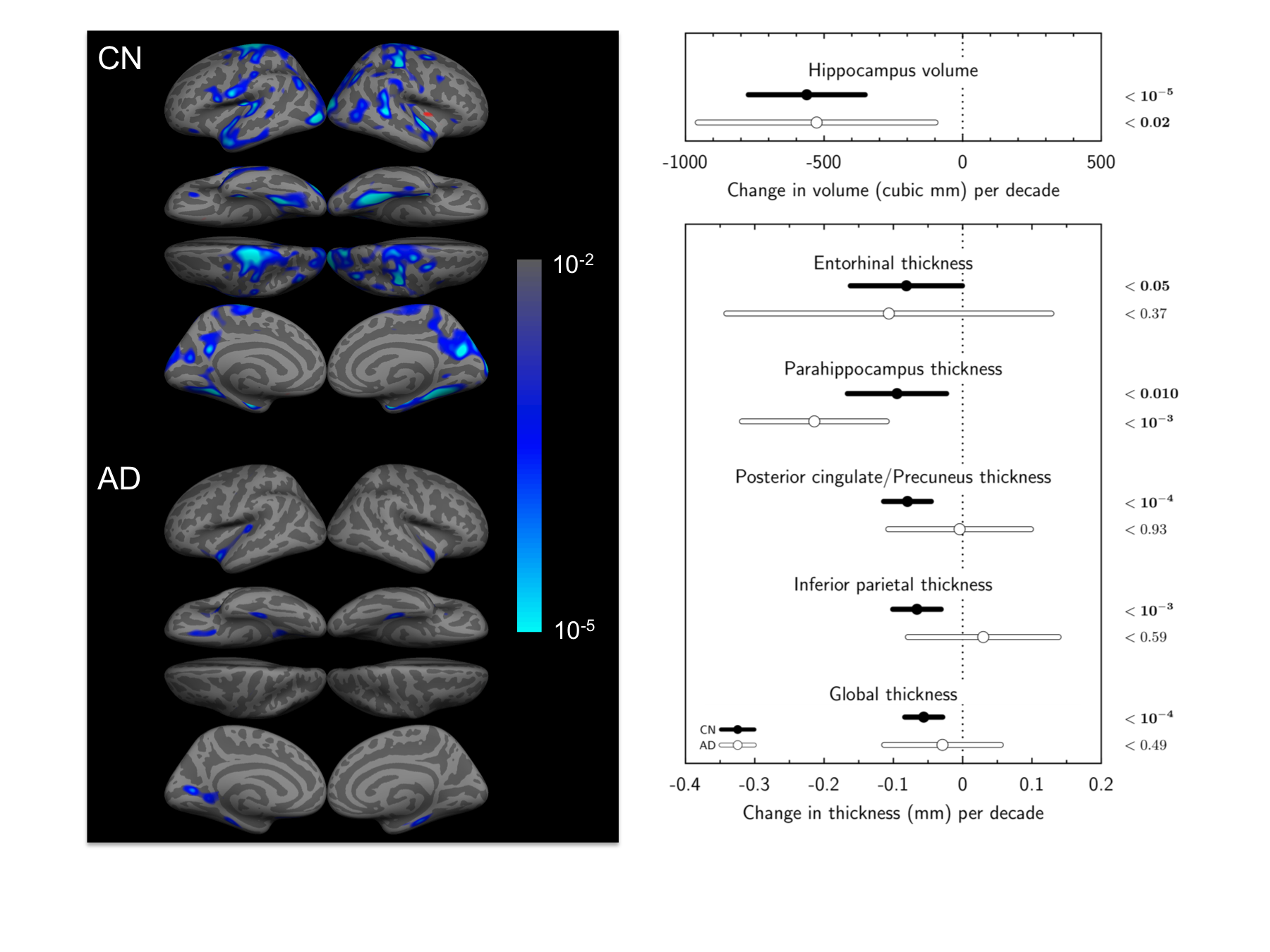
**

**Supporting Figure S6. Cortical thickness association with age, vertex-wise in CN group (upper left) and AD group (lower left), and by ROI in both groups (right).** Statistical significance (p-value) at each vertex of regression coefficient relating local cortical thickness and age (left panel); regression coefficients relating eTIV-adjusted hippocampal volume or regional cortical average thicknesses to age, and associated 95% confidence intervals and p-values (right panel).

**
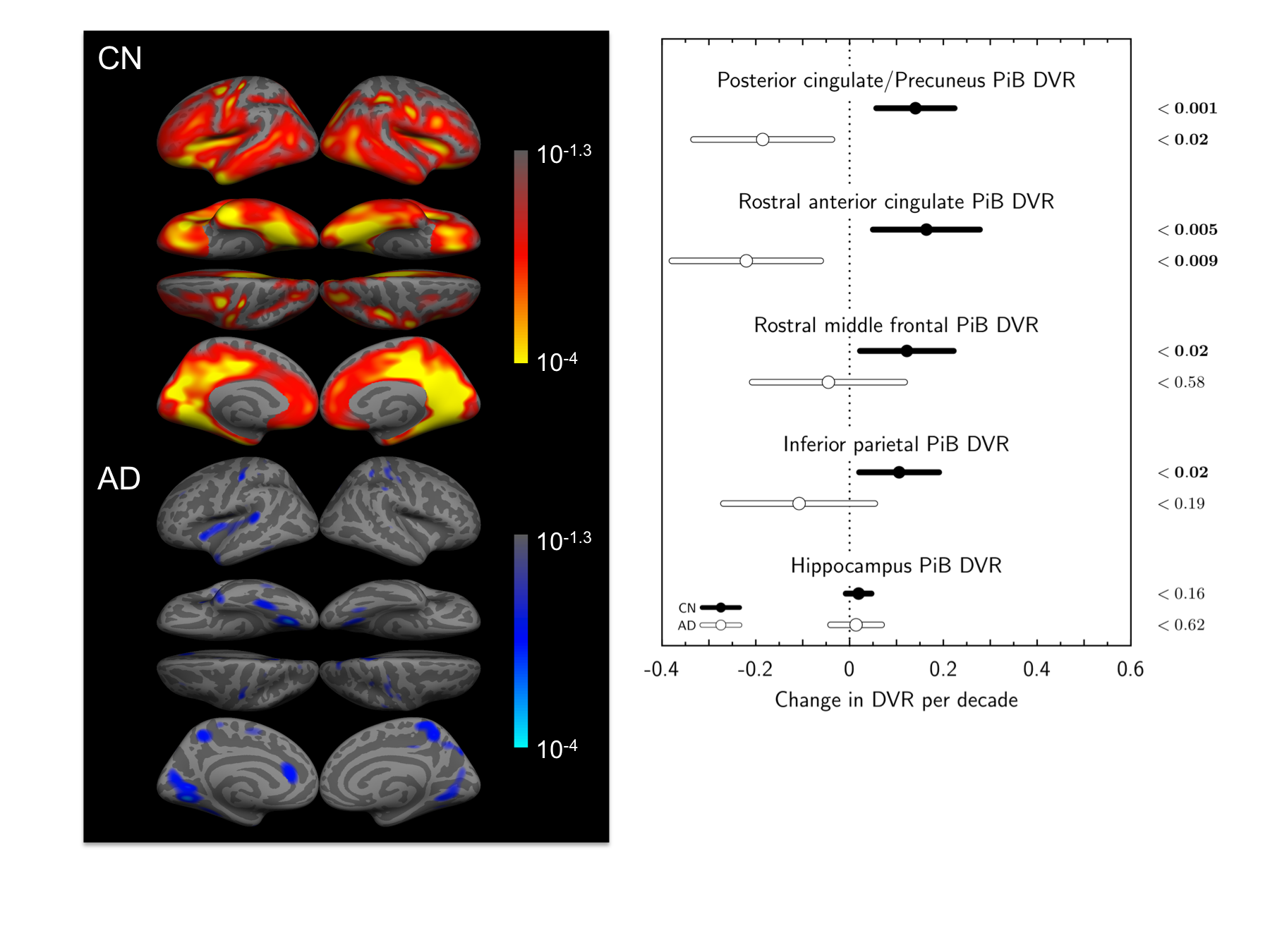
**

**Supporting Figure S7. PiB retention association with age vertex-wise in CN (upper left) and AD (lower left), and by ROI in both groups (right).** Statistical significance (p-value) at each vertex of regression coefficient relating local PiB retention (DVR) and age (left panel); red/blue indicates PiB retention increases/decreases with increasing age. (Right panel) regression coefficients relating change in regional average DVR per decade of age, and associated 95% confidence intervals and p-values.
